# Motility Disorders in Celiac Disease and Non-Celiac Gluten Sensitivity: The Impact of a Gluten-Free Diet

**DOI:** 10.3390/nu10111705

**Published:** 2018-11-07

**Authors:** Paolo Usai-Satta, Francesco Oppia, Mariantonia Lai, Francesco Cabras

**Affiliations:** 1 Gastroenteorlogy Unit, Brotzu Hospital, 09121 Cagliari, Italy; f.oppia@tiscali.it (F.O.); francescocabras@aob.it (F.C.); 2 Gastroenterology Unit, University of Cagliari, 09042 Monserrato, Italy; toninalai@medicina.unica.it

**Keywords:** celiac disease, non-celiac gluten sensitivity, gut motility, gluten-free diet

## Abstract

**Background:** There is evidence that digestive motor disorders are frequently present in untreated celiac disease (CD) patients. Similarly, non-celiac gluten sensitivity (NCGS) can be associated with gut motor disorders. In both cases, gut dysmotility can improve or be completely reversed with a gluten-free diet (GFD). **Methods:** A literature search for motility disorders in CD and NCGS patients was carried out using the online databases PubMed, Medline and Cochrane. **Results:** Esophageal, gastric, small bowel and gallbladder motor disorders are common in both children and adults with CD. Although the clinical consequences of these disorders are not clearly defined, gastric dysfunction could affect drug absorption and metabolism in the thyroid and neurological conditions associated with CD. The impact of a GFD on motility disorders is, however, controversial. No systematic studies are available on NCGS. NCGS frequently overlaps with irritable bowel syndrome (IBS) and similar pathophysiological mechanisms may be hypothesized. **Conclusions:** Mucosal damage may affect gut motility in untreated CD through perturbation of hormonal and neuro-immunomodulatory regulation. A persistent low-grade mucosal inflammation could explain the cases of persistent motor disorders despite a GFD. Further studies are needed to definitely assess the role of gut motor disorders in NCGS.

## 1. Introduction

Celiac disease (CD) is a permanent, chronic, gluten-sensitive disorder characterized by small intestinal mucosal injury and malabsorption in genetically predisposed individuals [1]. Self-reported wheat sensitivity without the diagnostic features of CD or wheat allergy has recently been named non-celiac gluten sensitivity (NCGS) [2]. Unlike CD, NCGS has no specific diagnostic test available. There is evidence suggesting that abnormal gut motility may frequently be present in CD and that all of the gastro-intestinal tract, including the gallbladder, can be involved. Several studies have shown that a gluten-free diet (GFD) can improve or normalize these disorders [3,4,5,6,7,8,9,10]. Not surprisingly, the data on motility disorders in NCGS are just emerging. In addition, a possible overlap between intestinal bowel syndrome (IBS) and gluten related disorders have been clearly demonstrated [11]. The aim of this review was to provide an overview of gut motility abnormalities in these gluten related disorders and to establish the real clinical impact of a GFD on these functional diseases.

## 2. Materials and Methods

A review of the literature on motility disorders in CD and NCGS patients was conducted using the online databases PubMed, Medline and Cochrane. Original research, reviews, and relevant books were included in the search. All the validated motility methods, manometry, pH-metry, gastric emptying studies (scintigraphy, octanoic acid breath testing and ecography), gallbladder ecography and lactulose breath testing for oro-cecal transit time, were included in this review.

## 3. Celiac Disease

### 3.1. Esophageal Motor Disorders

In two successive studies, using standard esophageal manometry, we found motor abnormalities in 67% of the adult patients examined [5,12]. These alterations consisted of nutcracker esophagus, a hypotonic lower esophageal sphincter (LES) and frequent repetitive (>3 peaks) contractions. Interestingly, 50% of celiac patients complained of dysphagia compared with only 9% of controls (*p* < 0.001) [12]. In addition, we performed esophageal pH-metry and the acid score was abnormal in 30% of celiac patients [5]. In 1998, Iovino et al. [13] confirmed that adult celiac patients with steatorrhea presented a higher prevalence of esophageal symptoms and a lower LES pressure when compared with celiac patients without steatorrhea and control subjects. Preliminary results from a more recent South American study demonstrated motility alterations in 83% of CD patients using esophageal manometry and pH-impedance [14].

### 3.2. Gastric Emptying Dysfunction

There is evidence suggesting that gastric emptying (GE) may frequently be delayed in CD patients, although a direct correlation to specific dyspeptic symptoms is not clear. To assess gastric emptying, different methods are available and they generally agree to demonstrate an altered gastric function in both children and adults with CD. In 1997, we documented delayed gastric emptying in 50% of the adult celiac patients examined in a scintigraphic study [5]. Similar results were observed by Perri et al. in a pediatric population of CD patients by using octanoic acid breath testing (OBT) [7]. Further studies using ecography and OBT confirmed that gastric emptying was constantly delayed in CD patients compared with that in healthy controls [8,9,10]. Recently, we performed OBT on a group of untreated adult celiac patients and 75% of them showed delayed gastric emptying with no significant correlation to specific dyspeptic symptoms [15].

### 3.3. Small Bowel Motility Disorders

Bassotti et al. [3] were the first to manometrically display fasting motor abnormalities, represented by discrete clustered contractions, giant jejunal contractions and bursts of non-propagated contractions in both adults and children with CD. Cucchiara et al. [4] evaluated 14 untreated pediatric CD patients by manometric study, and found that 90% had a reduced postprandial antral motility index, shorter activity fronts, prolonged small bowel phasic activity and uncoordinated peristalsis. In the study of 1997 we also performed a gastro-intestinal manometry to assess gastric and small bowel motility in CD patients [5]. During the fasting period 75% of celiac patients showed abnormal propagation of activity fronts and clustered contractions. The duration of antral contractile response to the standard meal was reduced compared with that of control subjects. A more recent study by Bassotti et al. confirmed that more than 80% of untreated celiac patients had discrete motor abnormalities of the upper gut in both fasting and fed periods [16].

### 3.4. Oro-Cecal Transit and Colonic Motor Disorders

Delayed oro-cecal transit in CD patients has been frequently demonstrated by the breath test method. Spiller et al. [17] first showed increased transit time in CD patients with steatorrhea compared with that in healthy controls. More recently, Chiarioni et al. [6] found delayed oro-cecal time in 16 adult CD patients by way of lactulose breath testing. Bai et al. [18] confirmed the results of oro-cecal transit while colonic transit measured by radiopaque markers (Metcalf method) showed faster transit times in untreated CD patients. In 2012, Benini et al. [19] showed that mouth-to-cecum transit time was more prolonged in CD patients than in controls. The same study performed colonic transit with radiopaque markers but no differences were found between celiac patients and controls. No studies are available on ano-rectal motility disorders in CD patients.

### 3.5. Gallbladder Motility

Gallbladder (GB) motility in CD patients has been studied since the 1970s. The scintigraphy performed in earlier studies and more recent ultrasonography agree upon delayed GB emptying in untreated celiac patients [20,21]. These abnormalities were associated with decreased peak plasma CCK levels and increased basal plasma somatostatin values. Benini et al. [19] performed a study to assess GB motility by means of ultrasonography and found that GB fasting volume and postprandial residual volume were significantly higher in CD patients than in controls.

### 3.6. Pathophysiological Mechanisms of Motility Disorders in CD Patients

It has been hypothesized that mucosal damage and inflammation may affect contractile gut motility through perturbations of the complex hormonal and neuro-immunomodulatory regulation of the intestinal mucosa. Low-grade mucosal inflammation and mast-cell infiltration could play an important role in untreated CD [22], as well as having been observed in patients with irritable bowel syndrome (IBS) [23]. Earlier studies showed that the secretion of several hormones regulating gastrointestinal motility could be altered as a consequence of intestinal mucosal damage [24,25]. A decrease in cholecystokinin and an increase in somatostatin have been implicated in gallbladder dysmotility, while an increase in neurotensin and plasma peptide Y levels has been suggested as a cause of delayed gastric emptying and esophageal abnormalities [9].

Another underlying mechanism for motor disorders in CD patients may relate to autonomic nervous system dysfunction [26]. In particular, we observed that extrinsic autonomic neuropathy could play a role in provoking upper-gut motor disorders in untreated CD patients [5].

### 3.7. Clinical Consequences of Motility Disorders in CD Patients

As already described dysphagia is a frequent complaint of celiac patients [12,13], although severe motor disorders and serious nutritional consequences have not been described in these cases. In addition, gastro-esophageal reflux may be more frequent in CD patients than in the general population [5], which could suggest a specific therapeutic approach. 

Surprisingly, a direct correlation to specific dyspeptic symptoms has not been demonstrated in the presence of delayed gastric emptying either in children or in adults with CD [5,7,10]. Similarly, small bowel dysmotility and altered oro-cecal transit have not been associated with specific clinical or nutritional problems.

On the other hand, special attention should be paid to the therapeutic implications of neurological conditions and thyroid dysfunction frequently associated with CD [27,28]. In these cases, gut dysmotility and, in particular, delayed gastric emptying could affect drug absorption and metabolism.

### 3.8. The Impact of GFD on Digestive Motor Disorders

Several articles have assessed the effects of a GFD on gut motor disorders, with conflicting results (Table 1). The majority of these studies showed that motor disorders can be completely reversible with a GFD.

Iovino et al. [13] showed that the prevalence of esophageal symptoms was significantly reduced in celiac patients after a year of a GFD. According to manometric evaluation, LES pressure was also significantly greater while on a GFD rather than on free diet.

Using OBT, Perri et al. [7] demonstrated that delayed gastric emptying could be normalized in the pediatric population afflicted by CD by means of a GFD. Similarly, a more recent OBT study showed that gluten withdrawal was effective in normalizing the gastric emptying time in all adult CD patients [10]. Benini et al. [8], using ultrasonography, showed that, after jejunal recovery, gastric emptying of the meal containing gluten remained unchanged, whereas emptying of the gluten-free meal was significantly shortened.

In a manometric small bowel study, four pediatric patients repeated the manometry after six months of a GFD and the traces had normalized [4]. Furthermore, a GFD normalized mouth-to-cecum transit in adult patients with CD in the study by Chiarioni et al. [6]. Finally, ecographic studies proved a GFD normalizes gallbladder motility [19,21].

On the other hand, we recently showed that delayed gastric emptying did not normalize on a GFD, despite the improved symptom score [15]. In addition, patients on a GFD showed motor abnormalities, albeit to a lesser extent than untreated CD subjects, in a small bowel manometric study [16]. In these patients, histological evaluation displayed the persistence of mild mucosal inflammation. Mouth-to-cecum transit time remained unchanged or more prolonged in CD patients than in controls after the introduction of a GFD in a lactulose breath testing study [19]. The same authors observed that duodenal infiltration with lymphocytes and mast cells remained higher than that in controls after gluten withdrawal.

Table 1 summarizes gut motility results in untreated and treated CD patients.

## 4. Non-Celiac Gluten Sensitivity (NCGS)

### Digestive Motor Disorders and NCGS

Although no systematic studies have assessed overall gastro-intestinal and gallbladder motor disorders in NCGS, some indirect evidences support the hypothesis of a possible derangement of digestive motor function in these patients [29].

Preliminary results by a pilot study suggest that patients with NCGS can present with colonic motility alterations that improve after implementation of a GFD [30].

The complex of symptoms associated with gluten-related disorders and in particular NCGS, such as diarrhea, constipation or abdominal pain, may overlap and be similar to those caused by irritable bowel syndrome with diarrhea (IBS-D) [11]. IBS complaints are often part of the NCGS clinical picture. For subjects diagnosed with NCGS, the ingestion of gluten exerts a direct effect on the onset of digestive symptoms and the exclusion of gluten is the treatment of choice. Only a portion of the patients with IBS relate their symptoms to a gluten-containing diet. In a randomized controlled trial, a gluten-containing diet provoked an increase in the number of bowel movements per day and higher small bowel permeability compared with a GFD in IBS-D patients [31]. No differences in overall gastro-intestinal motility were observed after one month of a GFD. Experimental data showed that transgenic mice, sensitized by gluten, had an altered barrier function and enhanced muscle contractility [32]. Furthermore, gluten-induced symptoms in IBS-D patients were associated with increased myosin light chain kinase activity and claudin-15 expression, as described in a recent trial [33].

On the other hand, it was recently demonstrated that gluten ingestion can exert objective effects on gastric and gallbladder motility in healthy subjects [34]. The potential role of proteins other than gluten on gastric and gallbladder motility has been hypothesized. In fact, other wheat components, such as amylase-tripsin inhibitors or fructans, have been linked to the development of gastrointestinal symptoms in both patients with NCGS and IBS [35,36].

## 5. Conclusions

Gut motility is frequently abnormal in untreated CD in both children and adults. Most pediatric studies address gastro-intestinal motor disorders and show similar findings to those of adult CD patients. These abnormalities may be independent of the presence and grading of symptoms. Special attention should be paid to possible nutritional and pharmacological consequences. In the majority of studies, a GFD improved or normalized these abnormalities. In the case of these disorders persisting while the patient is on a GFD, the presence of a chronic low-grade mucosal inflammation with permanent perturbation of the neuro-immunomodulatory regulation may be hypothesized.

Due to the absence of structured studies, data on motility disorders in NCGS are just emerging. The clinical picture of IBS can overlap with NCGS. Further studies are needed to definitely assess the role of gut motor disorders in NCGS.

## Figures and Tables

**Table 1:** Summary of more significant studies on gut motility in celiac disease (CD).

Authors [Ref]	Organs	Subjects	Methods	Findings	on GFD
Bassotti [3]	Small bowel	Adult and children CD	Manometry	Major frequence of migrating motor complex	No detected
Cucchiara [4]	Small bowel	Children CD	Manometry	Shorter activity front	Motor disorders normalized
Usai [5]	Esophagus, stomach, small bowel	Adult CD	Manometry	Nutcracker esophagus, esophageal reflux, delayed GE, abnormal small bowel activity front	No detected
			pH-metry		
			Scintigraphy		
Chiarioni [6]	OCT	Adult CD	Lactulose BT	Increased OCT	OCT normalized
Perri [7]	Stomach	Children CD	Octanoic BT	Delayed GE	GE normalized
Benini L [8]	Stomach	Adult CD	Ecography	Delayed GE	GE partially normalized
Rocco [10]	Stomach	Adult CD	Octanoic BT	Delayed GE	GE normalized
Usai [12]	Esophagus	Adult CD	Manometry	Hypotonic LES, repetitive waves	No detected
			pH-metry		
Iovino [13]	Esophagus	Adult CD	Manometry	Hypotonic LES	Increased LES pressure
Usai Satta [15]	Stomach	Adult CD	Octanoic BT	Delayed GE	Unchanged GE
Bassotti [16]	Small bowel	Adult CD	Manometry	Shorter activity front	Unchanged data
Spiller [17]	OCT	Adult CD	Lactulose BT	Delayed OCT	No detected
Benini F [19]	Gallbladder, OCT	Adult CD	Ecography	Delayed GB emptying and OCT	GB emptying normalized; OCT unchanged
			Lactulose BT		
Fraquelli [21]	Gallbladder	Adult CD	Ecography	Delayed GB emptying	GB emptying normalized

Notes: CD: celiac disease; GFD: gluten free diet; OCT: oro-cecal transit; LES: lower esophageal sphincter; BT: breath test; GE: gastric emptying; GB: gallbladder.
